# Oxidative Stress: A New Target for Pancreatic Cancer Prognosis and Treatment

**DOI:** 10.3390/jcm6030029

**Published:** 2017-03-09

**Authors:** Javier Martinez-Useros, Weiyao Li, Marticela Cabeza-Morales, Jesus Garcia-Foncillas

**Affiliations:** 1 Translational Oncology Division, OncoHealth Institute, Health Research Institute, University Hospital Fundación Jiménez Díaz-UAM, 28040 Madrid, Spain; weiyao.li@quironsalud.es (W.L.); jgfoncillas@gmail.com (J.G.-F.); 2 Facultad de Medicina, Universidad de Cartagena, Cartagena 2463, Colombia; marticelacabezamorales@gmail.com

**Keywords:** oxidative stress, pancreatic cancer, cytokines, interleukins, ROS, anti-oxidants, vitamins

## Abstract

Pancreatic ductal adenocarcinoma (PDAC) is one of the most lethal types of tumors, and its incidence is rising worldwide. Survival can be improved when tumors are detected at an early stage; however, this cancer is usually asymptomatic, and the disease only becomes apparent after metastasis. Several risk factors are associated to this disease. Chronic pancreatitis, diabetes, and some infectious disease are the most relevant risk factors. Incidence of PDAC has increased in the last decades. It is hypothesized it could be due to other acquired risk habits, like smoking, high alcohol intake, and obesity. Indeed, adipose tissue is a dynamic endocrine organ that secretes different pro-inflammatory cytokines, enzymes, and other factors that activate oxidative stress. Reactive oxygen species caused by oxidative stress, damage DNA, proteins, and lipids, and produce several toxic and high mutagenic metabolites that could modify tumor behavior, turning it into a malignant phenotype. Anti-oxidant compounds, like vitamins, are considered protective factors against cancer. Here, we review the literature on oxidative stress, the molecular pathways that activate or counteract oxidative stress, and potential treatment strategies that target reactive oxygen species suitable for this kind of cancer.

## 1. Introduction

Pancreatic ductal adenocarcinoma (PDAC) is the fourth leading cause of cancer death in the US. The number of new cases in 2016 is 53,070 and the number of deaths from this disease was 41,780 in the same year. Furthermore, it is estimated that it is the cause of ~227,000 deaths per year worldwide [1,2].

Survival can be improved when tumors are early detected. It has been reported that the five-year survival rate is 50% when tumors are <2 cm [3] and close to 100% for tumors <1 cm [4]. However, PDAC is usually asymptomatic, and the disease only becomes apparent after the tumor invades surrounding tissues or metastasizes to distant organs [5]. For the moment, surgical resection remains the best option to manage PDAC, and survival can be predicted based on the pathological characteristics of the tumor, such as T, N, and M stages, grade of differentiation, and positive resection margins [6].

There are some risk factors associated to PDAC initiation and development. Chronic pancreatitis causes a cumulative risk of 4% after 20 years [7]. Additionally, diabetes was recently considered a potential and early symptom of PDAC, as the disease was observed in approximately 30% of PDAC patients [8]. Furthermore, some infectious diseases that include *Helicobacter pylori* (HR = 1.5), Hepatitis B virus or Human Immunodeficiency virus have also been associated to this neoplasia [9,10]. Astonishingly, statistical reports carried out from 2003 to 2012 indicated that death rates due to PDAC are rising [11]. It is hypothesized that this increase could be reflected by the aging of the population in recent decades; however, observational studies have associated PDAC to some risk habits. The primary acquired risk factors for PDAC are cigarette smoking (HR = 1.74) and high alcohol consumption (HR = 1.1–1.5) [9,10]. Interestingly, other studies associated increased consumption of cooking and table salt with PDAC (*p* = 0.009 and *p* = 0.0001, respectively), and a similar correlation was found with smoked food (*p* < 0.01) [12]. Other observational studies link PDAC incidence to cadmium, arsenic, and lead exposure [13]. Indeed, countries with the highest levels of arsenic (more than 10 µg/L, values recommended by the World Health Organization [14]) are those with the highest incidence of PDAC. These countries include Baltic countries (especially Finland) and Central and Eastern European countries such as Austria, Czech Republic, Slovakia, and Hungary [15].

Another risk factor is obesity, determined by body mass index > 30 (HR = 1.2–1.5) [10,16]. Indeed, studies reported the high ability of adipose tissue to produce different pro-inflammatory cytokines, like IL-8, IL-6, or IL-2, and other enzymes, like lactate dehydrogenase (LDH) and tumor necrosis factor alpha (TNFα), that activate oxidative stress [17].

Oxidative stress is caused by the overproduction and cumulative production of free radicals in mitochondria, such as reactive oxygen species (ROS) that cause damage to lipids, proteins, and DNA [18]. Oxidative stress produces fatty-acid peroxidation whose metabolites possess very high toxicities and mutagenic properties. Some of the most important compounds are 4-hydroxy-2-nonenal (4-HNE) [19] and malondialdehyde (MDA) [20]. Both compounds produced in the adipose tissue have an extraordinary effect on whole body metabolism.

In contrast, vitamin intake was associated with reduced levels of M1-dG (pyrimido(1,2-a)purin-10(3H-)one) [21], a resultant compound of physiological reaction between MDA and several nucleosides. This fact supports the role of vitamins as a protective factor against cancer.

The present systematic review collects and analyses the role of oxidative stress factors associated to PDAC, and their potential uses as targets for future designed therapies.

## 2. The Oxidative Stress in Cancer

ROS species produced by oxidative stress have been detected in various cancers due to their high metabolic activity, and they may promote many aspects to maintain the aggressive phenotype [22].

Oxidative stress is produced by a change in the equilibrium between ROS and anti-oxidant compounds. When this balance is disturbed in support of the oxidants, oxidative stress occurs. However, cancer cells have the ability to maintain ROS levels to avoid cell death [23].

Internal oxidative stress is supported by peroxisomes and some enzymes, particularly detoxifying enzymes from the P450 complex, xanthine oxidase, and the nicotinamide adenine dinucleotide (NADPH) oxidase complexes, which include the NOX family [24].

In fact, NADPH oxidase is a major source of intracellular ROS in pancreatic cancer cells [25]. Most of these enzymes act in the mitochondria, which is the main organelle involved in oxidative stress [26,27]. Reactive species can be classified into four groups based on the main atom involved: reactive oxygen species (ROS), reactive nitrogen species (RNS), reactive sulfur species (RSS), and reactive chloride species (RCS) [26]. The most important compound from all of the above are ROS because they are the most abundantly produced by cell metabolism.

Oxidative stress affects key signaling proteins involved in several molecular pathways, such as nuclear factor erythroid 2-related factor 2 (NRF2), kelch-like protein 19 (KEAP1), mammalian target of rapamicin (mTOR), c-MYC, P53, and protein kinase C (PKC), and other proteins involved in mitogen-activated protein kinase (MAPK) such as RAS, RAF, extracellular-regulated kinase 1/2 (ERK1/2), mitogen-activated protein kinase kinase (MEK), c-Jun N-terminal kinase (JNK), and P38 [28,29]. P38 acts as a key sensor of oxidative stress, and its redox sensing function is essential in the control of tumor development blocking proliferation or promoting apoptosis [30]. Among them, NRF2 is considered to be the master regulator of the anti-oxidant response [31].

Various factors, including ROS, pro-inflammatory cytokines, other growth factors, and extracellular matrix proteins, are involved in the instability of PDAC [32,33]. However, ROS and pro-inflammatory cytokines are considered to be the most important factors involved in the pathogenesis of PDAC.

### 2.1. ROS

**Figure 1:**
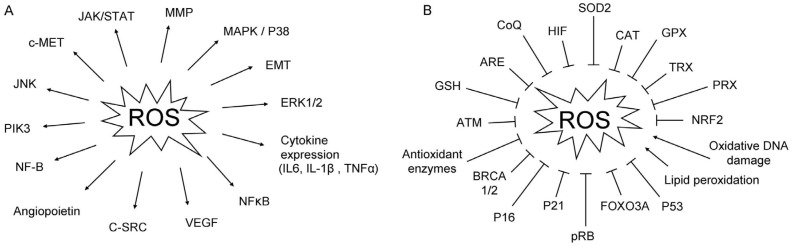
Molecular factors and pathways involved in reactive oxygen species (ROS) regulation. (**A**) Different genes, and pathways activated by ROS; (**B**) different genes, enzymes, and biochemical reactions that inhibit or promote ROS production. Arrows indicate activation and bar-headed arrows refer to inhibition.

ROS can promote several pathways that enable tumor progression and aggressiveness through regulation of proliferation, apoptosis, and invasion of tumor cells (Figure 1A). These pathways include: cellular proliferation through MAPK, especially through ERK1/2 and NF-κB [34,35]; evasion of apoptosis by regulation of c-SRC, NF-κB, PIK3/AKT, and JAK/STAT (Janus kinase/signal transducer and activator of transcription) [25]; tissue invasion and metastasis by modulation of metalloproteinases (MMP) into the extracellular matrix; c-MET overexpression and Ras homolog gene/Ras-related C3 (RHO-RAC) interaction; and angiogenesis through the release of vascular endothelial growth factor (VEGF) and angiopoietin [26].

Different signaling pathways are directly involved in tumorigenesis and can also regulate ROS production (Figure 1B). Glutathione (GSH) is a crucial factor to protect cells from oxidative compounds. Elevated levels of GSH are observed in several types of cancer, and has been associated to chemotherapy resistance [36].

Apart from the protection provided by specific anti-oxidant enzymes, such as superoxide dismutase (SOD), catalase (CAT), glutathione peroxidases (GPXs), thioredoxins (TRXs), and peroxiredoxins (PRXs), the master regulator of the anti-oxidant response is the transcription factor NRF2. ROS levels are tightly controlled and predominantly regulated by NRF2 [37]. Initially, it was thought that NRF2 was able to regulate oxidative stress levels through modulation of anti-oxidant response elements (ARE). Activation of NRF2 is dependent on some kinase pathways, such as MAPK and PIK3 [38,39]. In addition, NRF2 modulates the expression of hundreds of genes, including not only anti-oxidant enzymes, but also a large number of genes that control several processes, including immune response, inflammatory cascade, tissue remodeling and fibrosis, carcinogenesis, and metastasis [40]. A preclinical study with a murine model that lacks NRF2 developed more severe intestinal inflammation and aberrant crypts that suggested its role as a protective factor to prevent inflammation and carcinogenesis [41].

In normal cells, several tumor suppressor genes counteract oxidative stress and equilibrate the redox balance by prevention of lipid peroxidation and oxidative damage to DNA and protein. One of the mechanisms to achieve this protective effect is hypomethylation of anti-oxidative genes, but also upregulation of pro-apoptotic genes such as *TP53*, *FOXO*, Retinoblastoma (*RB*), *P21*, *P16*, and breast cancer susceptibility genes 1 and 2 (*BRCA1* and *BRCA2*) [42]. ROS removal is crucial to avoid cell death, especially dealing with elevated ROS levels in cancer cells. In the absence of wild-type tumor suppressor genes, like *TP53*, cancer cells switch off several anti-oxidative pathways and leads to ROS accumulation [43]. Here, oncogenes play an important role in the control of ROS balance. One of the most important pathways related to oxidative stress and cancer is the MAPK pathway. The *RAS* gene family is a key activator of this pathway. This family encodes three proteins, H-, N-, and K-RAS, which participate in extra-cellular signaling [44]. Mutation on *RAS* results in an upregulation of ROS levels, contributing to the DNA damage and malignant transformation [45]. The upregulation of other oncogenes, like *RAF*, *MYC*, or Cyclin E1 (*CCNE1*), could silence the effect of tumor suppressor genes that cooperate to increase ROS production [46]. Moreover, ROS is able to induce epithelial-mesenchymal transition (EMT) factors [47].

The role of ROS in PDAC is a doubled-edged sword that depends on the concentration in cells. ROS facilitates cancer progression and promotes the malignant phenotype in mild-to-moderate levels, while over-production of ROS damages cancer cells dramatically and leads to cell death. It is known that cancer cells are characterized by a moderate rate of ROS production, which enhances the tumor metabolic adaptation, proliferation, survival, and angiogenesis [48].

In PDAC the increased ROS production is considered a hallmark, thus, it is thought to be both a pro-survival and an anti-apoptotic factor in this type of tumor [49]. In contrast, a study associated lower levels of ROS with resistance to gemcitabine and other chemotherapies in PDAC cells [50].

Hypoxia is a characteristic feature of PDAC, thus, hypoxia-inducible factor (*HIF*) has been reported to be promoted by ROS through the PIK3/AKT pathway [51]. Moreover, ROS acts as an adaptive strategy to inhibit autophagy and this effect may be mediated by upregulation of the AKT/mTOR (mammalian target of rapamycin) pathway [52]. Here, ROS has a dual role in carcinogenesis and it is able to promote chemosensitivity through mTOR inhibition [52].

### 2.2. ROS and microRNAs Regulation

MicroRNAs (miRNA or mir-RNA) are small non-coding, evolutionarily-conserved RNA molecules of about 21–24 nucleotides, which regulate gene expression that cause a translational repression or mRNA cleavage. This fact depends on the partial or complete base complementarity with the 3′-untranslated region (UTR) of target messenger RNAs [53]. MiRNA expression is associated with several cellular processes, including cancer. ROS species produced by ionizing radiation, etoposide, and peroxide accumulation in the extracellular matrix has been demonstrated to induce alterations in miRNA expression patterns [54].

Several ROS-related miRNAs have been described and associated with cancer development [55]. In PDAC, overexpression of miRNA-155 inhibits FOXO3a, leading to a decrease of SOD2 and CAT, which induce ROS accumulation in cancer cells [56].

MiR-128a is downregulated in cancer and its re-expression is able to arrest proliferation by BMI1 downregulation, which changes the redox equilibrium by an increase of ROS in medulloblastoma tumor cells [57]. Another study showed the role of miR-200a and miR-141 as regulators of oxidative stress response in high-grade human ovarian carcinomas, the most lethal gynecologic malignancy [58]. ROS levels derived from NADPH oxidase have been shown to be involved in the proliferation and invasiveness through regulation of miRNA-21 in prostate cancer cells [59].

### 2.3. Polimorphisms Associated to Oxidative Stress

Some studies associated single nucleotide polymorphisms (SNPs) found in oxidative stress genes to cancer.

A pancreatic cancer study performed with 189 patients and 486 controls, showed that individuals with *SOD2* polymorphism (rs4880) had 43% lower risk than those who were homozygous for the wild-type allele (HR = 0.57; CI = 0.37–0.89). However, the selected genetic variants of *CAT* and *XRCC1* (X-ray repair cross-complementing group 1) neither influenced the risk of pancreatic cancer [60]. Another study performed with 500 patients did not find any association between PDAC risk and polymorphisms in the oxidative stress-modifying genes: superoxide dismutase (*SOD2* (Ala16Val, rs4880), *SOD3* (Arg231Gly, rs1799895), nor in nicotinamide adenine dinucleotide phosphate quinone oxidoreductase (*NQO1* (Pro187Ser, rs1800566) and *NQO2* (Phe47Leu, rs1143684)) [61].

C677T polymorphism on *MTHFR* (methylenetetrahydrofolate reductase) is a key enzyme in folate metabolism, conferring susceptibility to chronic pancreatitis [62]. Human paraoxonase 1 (PON1) has been associated to a decreased levels of systemic oxidative stress. Although there was no correlation between polymorphisms of the *PON1* gene and PDAC tumor stages or other clinical parameters, significant association was found with clinically-relevant malnutrition [63].

### 2.4. Inflammatory Cytokines and ROS Accumulation in PDAC

ROS induces pro-inflammatory cytokine expression that, in most instances, are involved in EMT [64], and also ROS production is exacerbated by feedback by pro-inflammatory cytokines in cancer cells (Table 1) [65]. The release of superoxide, hydrogen peroxide, and hydroxyl radicals by macrophages and neutrophils is maintained by NADPH activation of the plasma membrane [66]. In addition, there are many other factors that can stimulate cancer cells to generate ROS, such as insulin-like growth factor I (IGF1), and fibroblast growth factor-2 (FGF2) [25].

Cytokines promote PDAC progression through modulation of the tumor microenvironment. They also act directly on proliferation, invasion, and metastasis. Cytokines are produced by leukocytes, stellate cells, and adipocytes. Several pro-inflammatory cytokines, and other compounds, that are associated with oxidative stress, were detected in the serum of different pancreatic diseases such as interleukin-2 (IL-2), IL-6, IL-1β, IL-8, TNF-α, TGF-β, lactate dehydrogenase (LDH), 4-hydroxynonenal (4-HNE), and malondialdehyde (MDA) [67,68,69,70,71,72].

IL-2 and IL-6 induce the expression of vascular endothelial growth factor (VEGF) in PDAC cells and stimulate angiogenesis and tumor vascularization [73]. Furthermore, IL-6 induces phosphorylation of STAT3 that promotes PDAC proliferation and inhibits autophagy [74].

PDAC invasiveness is also supported by IL-1β. One study reported that IL-1β is able to induce NF-κB and expression of cyclooxygenase-2 (COX2) that conferred chemoresistance [75].

IL-8 stimulates the expression of VEGF, VEGF receptor, and neuropilin-2, which are key molecules in angiogenesis. In addition, IL-8 increases the activation of MAPK pathway to promote cell growth, survival, and tumorigenesis [67]. It has been reported that IL-8 promotes aggressiveness and invasiveness in PDAC by regulation of MMP2 activity [76].

TNF-α promotes PDAC proliferation, induces the invasiveness of human PDAC cells and promotes tumor growth and metastasis in mice models. This pre-clinical research suggested anti-TNF-α therapy as an alternative to suppress tumor growth and metastasis in PDAC [77]. TGF-β also induces proliferation and invasiveness in PDAC through the matrix metalloproteinase-2 (MMP-2) and urokinase plasminogen activator system [78,79]. LDH production has been enhanced by increased activity of Myc and HIF in human cancers [80]. LDH has been considered a prognostic biomarker of advanced disease in 196 recruited PDAC and a predictive biomarker for gemcitabine-based chemotherapy (*p* = 0.04) and hepatic metastasis (*p* = 0.01). In addition, LDH levels were associated with shorter survival (*p* = 0.001) [81]. Another study with 291 patients showed an association between LDH levels >250 U/L and progression-free survival (*p* = 0.004) and overall survival (*p* < 0.001), but only in univariate analyses [82].

The more influential products of oxidative stress are 4-hydroxy-2-nonenal (4-HNE) and malondialdehyde (MDA). Both compounds are produced in the adipose tissue and have an extraordinary effect on whole body metabolism [83].

4-hydroxynonenal (4-HNE) is a biomarker of oxidative stress and an important player that mediates a high number of signaling pathways. 4-HNE forms covalent adducts with nucleophilic functional groups in proteins, nucleic acids, and membrane lipids. Macromolecular adducts formed in mitochondria and associated to inflammation are involved in the initiation and progression of PDAC [70,84]. Overexpression of 4-HNE has also been found in the gallbladder epithelium from patients with pancreaticobiliary malfunction compared to normal gallbladders (*p* < 0.05) [85]. In contrast, while 4-HNE at low concentrations can protect cancer cells against damage, at high concentrations cells undergo apoptosis, or even necrosis. Thus, 4-HNE formation may provide a therapeutic value to prevent or treat cancer [86].

MDA regulates islet glucose-stimulated insulin secretion through the WNT pathway [87]. Under stress conditions, MDA has high ability to react with proteins or DNA, which leads to the formation of adducts [88]. In PDAC the inflammatory conditions have been associated with elevated levels of MDA (*p* = 0.048) [72], and it has also been proposed as a predictive biomarker of response to pro-apoptotic drugs, such as indole-3-acetic acid and horseradish peroxidase [89].

## 3. Therapies against Oxidative Stress

The inhibition of oxidative stress is currently a target to inhibit tumor growth in different types of cancer. The neutralization of ROS, oxidative stress-related cytokines, and other pro-inflammatory factors by different agents has been used to decrease the effects of oxidative stress in disease. These agents are based not only on different treatments as natural or artificial anti-oxidants, but also could be based on changes in diet [90]. Some of the agents used as anti-tumoral therapies include vitamin E, vitamin C, curcumin, and coenzyme Q10.

Vitamin E includes four subtypes of tocopherols and other four tocotrienols, which are known for their anti-oxidant properties. They inhibit mutant *KRAS*-driven pathways, such as MEK/ERK, PIK3/AKT, NF-κB/P65, BCL-XL, and induced P27 in PDAC. δ-tocopherol also triggers apoptosis cascade through activation of BAX and caspase 3 by an increase in plasma levels of CK18 in PDAC [91]. In this pre-clinical study, vitamin E was able to interact with oncogenic KRAS; thus, it has been proposed as an anti-tumor agent [91]. The efficacy of vitamin E has been evaluated at different daily doses (from 200 to 3200 mg) over 13 days before surgery and one dose on the day of surgery. The results showed evidence of a significant induction of apoptosis in tumoral cells with an increased in the levels of cleaved caspase-3 [92]. Patacsil et al., reported that vitamin E could inhibit cell proliferation and induce apoptosis in PDAC-derived cells lines through downregulation of Survivin and XIAP (X-linked inhibitor of apoptosis proteins) [93].

Vitamin C, also known as ascorbic acid or formulated as ascorbate sodium salt, has been one of the mostly used agent in cancer therapies, especially to counteract oxidative stress. Vitamin C allows the production of H_2_O_2_ in the extracellular fluid surrounding tumor that kills cancer cells [94,95]. Another mechanism associated to vitamin C is the disruption of the Warburg effect in tumor cells with the *KRAS* mutant genotype through downregulation of key metabolic checkpoints [96]. Vitamin C induces RAS unbinding from ERK1/2, and PKM2 phosphorylation. Thus, it leads to a strong downregulation of glucose transporter 1 (GLUT-1), which causes a high blockage of the Warburg effect [97]. The safety of vitamin C is one of its most notable characteristics. In vitro studies have shown no toxicity for normal cells, even with concentrations > 20 mM, however tumor cells die at concentrations < 10 mM [96].

In preclinical studies, vitamin C has been associated with a decrease of viability in PDAC-derived cell lines. Moreover, untransformed cells were unaffected by 20 mM ascorbate [98]. The susceptibibility of PDAC-derived cell lines to ascorbate was also demonstrated by Du et al. at concentrations of 5 and 10 mM [99]. Nevertheless, the route of administration of vitamin C is rather controversial because oral administration of ascorbate (1 g/kg) increased plasma concentration to 50 μM; however, the same dose administered intraperitoneally increased plasma concentration to 15 mM [100].

The combination of vitamin C with gemcitabine and erlotinib in patients with metastasic PDAC did not increase toxicity and kept most of the patients with stable disease [101]. Welsh et al. also used ascorbate with gemcitabine, in patients with metastasic and node-positive PDAC, and achieved similar results [102]. Interestingly, high doses of ascorbate parenterally, in combination with conventional chemotherapy, enhanced chemosensitivity and reduced toxicity of chemotherapy in patients with ovarian cancer [103].

Another natural anti-oxidant is curcumin, a plant-derived natural polyphenol that has been used in some studies with PDAC. The administration of 8 g of curcumin orally had antitumor activity in this kind of patient [104,105]. However, the plasma curcumin levels remain low despite high administration doses. Recently, curcumin has been reformulated in nanoparticles with longer half-lives that have increased plasma concentration; thus, it could be a promising tool to be used in patients [106].

Coenzyme Q10 (CoQ, or ubiquinone) is a lipid that acts in mitochondria as an electron shuttle between complexes I and, II, and complex III of the respiratory chain. CoQ is also a cofactor for other dehydrogenases and an essential anti-oxidant [107]. It has been reported that a deficit of CoQ decreases ATP production with a subsequent antitumor activity [107]. Hertz et al. evaluated the efficacy of CoQ in combinations with other vitamins as chemotherapy in end-stage cancer patients. This study showed an improved survival without any toxicity [108].

Table 2 summarizes anti-oxidant molecules used in PDAC treatment.

Consideration must be given to PDAC that has the highest incidence of *KRAS* mutation of all types of tumors, and more than 50% of patients could exhibit this abnormality [109,110]. Additionally, the *KRAS* mutation is considered a critical event for the initiation of this type of cancer [111]. The fact that vitamins, like vitamin E and vitamin C, enable the inhibition of mutant *KRAS*-driven pathways via MAPK or PIK3/AKT offers new treatment strategies for this kind of tumor for which few therapies are available.

On the other hand, the clinical applicability of anti-oxidants is reinforced by the ability to trigger the apoptosis cascade selectively on tumor cells, but not on healthy cells. Then, there is another and even more important question about whether a combination of anti-oxidants alone or in combination with chemotherapy will improve patients’ survival. Preclinical models have proved the efficacy of anti-oxidants; however, clinical trials have mainly focused on the safety of anti-oxidants alone or combined with standard chemotherapy. Therefore, further randomized controlled trials with higher sampling are needed to introduce these compounds in routine clinical practice.

## 4. Conclusions

PDAC is one of the most deadly cancers worldwide, and despite new methods of early diagnosis, surgery, and drug discovery, tumor cells metastasize to different organs, thereby reducing survival significantly. Furthermore, the fact that PDAC response to chemoradiotherapy is poor makes this disease one of the most challenging cancers. Some of the factors associated with the increasing incidence of pancreatic cancer are also factors related to ROS overexpression. Obesity, cigarette smoking, high alcohol intake, and inflammatory processes are the most relevant factors [112]. If ROS is not counteracted by anti-oxidants, they can cause damage to lipids, proteins, and DNA [18]. This damage leads to toxic and mutagenic metabolites that alter cell homeostasis. Ultimately, cancer is based on DNA aberrations and the transformation to neoplasic phenotypes is driven by genetic changes; however, ROS production enables tumor cells to acquire invasive and aggressive phenotypes. Vitamins have a protective factor against cancer and have been proposed as new chemotherapeutic treatment. However, the effectiveness of anti-oxidants as cancer treatments is questionable and the results are inconclusive.

The strategies to modulate oxidative stress in cancer with anti-oxidants could be, in the future, an effective approach due to their null toxicity and safety profile. Therefore, these molecules could represent a basis for future drug design to fight against PDAC.

On the other hand, in clinical trials anti-oxidants are normally tested in combinations with other chemotherapies, where their effect could be veiled by the other drug. However, the option to test anti-oxidants as single agents in clinical trials could be more difficult to obtain funding to carry out such studies. What is true is the significant potential use of anti-oxidants to increase the chemosensibility of standard chemotherapy; this fact will enhance, in the near future, the interest on them, and on their combinations.

## Figures and Tables

**Table 1:** Inflammatory cytokines and other factors involved in the pathogenesis of pancreatic ductal adenocarcinoma.

Factor	Target	Role in Tumorogenesis	References
NADPH	H_2_O_2_, -OH, O_2_-radicals	loss of biochemical homeostasis	[66]
IGF1	Increased ROS production and NAD(P)H oxidase activity	anti-apoptosis and agresiveness	[25]
FGF2	Increased ROS production	anti-apoptosis	[25]
IL-2	VEGF	angiogenesis	[73]
IL-6	STAT3	proliferation	[74]
IL-1β	NF-κB, COX2	invasiveness, chemoresistance	[75]
IL-8	VEGF, VEGFR, Neuropilin-2, MAPK, MMP2	proliferation, invasiveness, survival angiogenesis	[67,76]
TNF-α	NF-κB, AP1	invasiveness	[77]
TNF-β	MMP2, urokinase	proliferation, invasiveness	[78,79]
LDH	Regulated by c-Myc and HIF1	predictive biomarker of gemcitabine response, prognosis	[80,81,82]
4-HNE	GSH	inflammation, pancreatic maljunction	[70,84,85,86]
MDA	DNA, WNT pathway	inflammation, apoptotic biomarker	[72,87,88,89]

**Table 2:** Anti-oxidant therapies in PDAC.

Molecule	Dose	Study	*n*	Parameters	Results	Reference
Vitamin E	200 mg/kg twice a day, for 12 months	In vivo	92 mice	Survival, progression	Increased survival (*p* < 0.025). Induced BAX and Caspase 3	[91]
Vitamin E	200–3200 mg daily for 13 days	Phase I	25 patients	Safety, pharmacokinetics, apoptosis	Apoptosis induction (*p* = 0.044)	[92]
Vitamin E	25.1 to 51.3 μM	In vitro	PANC-1, COLO-357, and ASPC-1 cell lines	Cell viability, apoptosis, cell cycle	Inhibition of proliferation. Apoptosis induction (*p* < 0.01).	[93]
Curcumin	8 g orally daily	Phase II	25 patients	Tumor volume and interleukin levels	Decreased pSTAT3 (*p* = 0.009), COX2 (*p* = 0.029), and L-6, IL-8, IL-10, and IL-1RA (- to 35-fold)	[105]
Ascorbate	Ascorbate dose of 15 g was infused with subsequent dose escalation of 25 to 100 g over 50 min/0–20 mM for 1 h	In vivo	194 mice	Tumor volume and ascorbate levels	Ascorbate decreased growth of ovarian (*p* < 0.005), pancreatic (*p* < 0.05), and glioblastoma (*p* < 0.001) mice tumors	[96]
Ascorbate	50.75 and 100 g three infusions per week, for eight weeks	Phase I	9 patients (stage IV)	Safety and progression	Null toxicity. Seven patients with stable disease, 2 patients with progression disease	[101]
Ascorbate	4 g/kg for two weeks 0.5–10 mmol/L for 1 h	In vivo	28 mice	Tumor growth	Ascorbate inhibited tumor growth (*p* = 0.001)	[99]
Ascorbate	15–125 g twice weekly	Phase I	9 patients	Safety and progression	Ascorbate combined with gemcitabine should be safe and well tolerated	[102]

Note: *n*: number of participants.

## Abbreviations

ARE	Anti-oxidant Response Elements
BCL-XL	B-cell lymphoma-extra large
BRCA	Breast cancer susceptibility genes
CAT	Catalase
CCNE1	Cyclin E1
CI	Coefficient interval
CK	Cytokeratin
CoQ	Coenzyme Q10
COX2	Cyclooxygenase-2
EMT	Epithelial-mesenchymal transition
ERK	Extracellular-regulated kinase
FGF-2	Fibroblast growth factor 2
GLUT-1	Glucose transporter 1
GPXs	Glutathione peroxidases
GSH	Glutation
HIF	Hypoxia-inducible factor
4-HNE	4-Hydroxynonenal
HR	Hazard ratio
IGF1	Insulin-like growth factor I
IL	Interleukin
KEAP1	Kelch-like protein 1
LDH	lActate dehydrogenase
MAPK	Mitogen activated protein kinase
M1-dG	Pyrimido 1,2-a purin-10 3H- one
MDA	Malondialdehyde
MEK	Mitogen-activated protein kinase kinase
MMP	Metalloproteinases
MTHFR	Methylenetetrahydrofolate reductase
mTOR	Mammalian target of rapamicin
NADPH	Nicotinamide adenine dinucleotide
NQO1	Nicotinamide adenine dinucleotide phosphate quinone oxidoreductase
NRF2	Nuclear factor erythroid 2-related factor 2
PDAC	Pancreatic ductal adenocarcinoma
PIK3	Phosphoinositide 3-kinase
PKC	Protein kinase C
PKM2	Pyruvate kinase isozymes M2
PON1	Human paraoxonase 1
PRXs	Peroxiredoxins
RB	Retinoblastoma
RCS	Reactive chloride species
RHO-RAC	Ras homolog gene/Ras-related C3
RNS	Reactive nitrogen species
ROS	Reactive oxygen species
RSS	Reactive sulfur species
SNPs	Single nucleotide polymorphisms
SOD	Superoxide dismutase
TNFα	Tumor necrosis factor alpha
TRXs	Thioredoxins
UTR	Untranslated region
VEGF	Vascular endothelial growth factor
XIAP	X-linked inhibitor of apoptosis proteins
XRCC1	X-ray repair cross-complementing group 1
